# Ultrasonically Fabricated Beta-Carotene Nanoemulsion: Optimization, Characterization and Evaluation of Combinatorial Effect with Quercetin on Streptozotocin-Induced Diabetic Rat Model

**DOI:** 10.3390/pharmaceutics15020574

**Published:** 2023-02-08

**Authors:** Manohar Mahadev, Akhilesh Dubey, Amitha Shetty

**Affiliations:** 1 Nitte (Deemed to be University), NGSM Institute of Pharmaceutical Sciences, Department of Pharmaceutics, Deralakatte, Mangalore 575018, India; 2 Department of Pharmaceutics, JSS College of Pharmacy, JSS AHER, Mysuru 570015, India

**Keywords:** ultrasonication, Box-Behnken design, diabetes mellitus, beta-carotene, quercetin, nanoemulsion

## Abstract

Diabetes mellitus (D.M.) is a metabolic disease that has affected over 500 million people globally. Bioactive compounds such as β-carotene and Quercetin have gained research interest for their potential antidiabetic properties, and bioactives have reported superior combinatorial effects in several ailments, including D.M. However, poor oral bioavailability has limited their potential application. Thus, the present study was focused on developing ultrasonically fabricated β-Carotene nanoemulsion (βC-NE) by employing capmul as the oil phase, Gelucire 44/14 as surfactant and Acconon MCM C8 as co-surfactant. The 3 factor- 3 level Box-Behnken design (BBD) was applied to optimise the βC-NE and study the impact of selected independent variables such as % Smix (5 to 9%), amplitude (20–30%) and sonication time (2.5–7.5 min) on responses including globule size (G.S.), poly dispersibility Index (PDI) and entrapment efficiency (E.E.). Further, the combinatorial effect of βC-NE with Quercetin Nanoemulsion (QU-NE) in the streptozotocin-induced diabetic rat model was evaluated. The results exhibited that 7% Smix at 25% amplitude for 5 min produced βC-NE with a droplet size of 153.1 ± 12.25 nm, 0.200 ± 0.04 PDI, and 73.25 ± 3.25% E.E. The βC-NE showed superior in-vivo bioavailability by 5.38 folds. The βC-NE, combined with QU-NE, exhibited potential therapeutic benefits in controlling body weight, blood sugar level, lipid levels, and tissue damage markers. Additionally, the pancreatic cells and hepatic cells were well protected. These results demonstrate the potential benefits of βC-NE and QU-NE in combination and recommend them as a substitute strategy for diabetes.

## 1. Introduction

The development of promising medicinal agents to inhibit or impede the inception of Diabetes Mellitus (D.M.) and its complications are of utmost clinical significance in contemporary medicine. Polyphenol bioactive compounds have been extensively utilised to retard D.M. for potential health benefits and low toxicity [1]. Extensive clinical investigations specified that some dietary bioactive compounds, including flavonoids and carotenoids, could influence possible cellular mechanisms and aid in managing D.M. by considering their value as novel therapeutic agents are encouraging in modern novel studies.

The β-carotene (β-crt) is an antioxidant and acts as a scavenger of lipophilic radicals within the membranes of cell compartments; hence, β-crt may protect against type 2 diabetes by inhibiting lipid peroxidation in cell membranes. A study confirmed that beta carotene could modulate the metabolisms of lipid and carbohydrates and improves the activity of ß-pancreatic cells, thereby improving hyperglycaemic conditions [2]. It has also been studied and reported to regulate the functions of β- cells of the pancreas leading to insulin secretion, thereby alleviating oxidative and inflammatory stress [3].

Whereas, Quercetin (Q.U.) has been reported that approximately 80% of glucose uptake transpires via the human body through insulin-sensitive skeletal muscles. Hence, deterioration in glucose uptake in skeletal muscles can lead to the pathogenesis of type 2 diabetes by changing whole-body glucose homeostasis [4]. Quercetin induces AMPK activity in skeletal muscles and inhibits glucose 6 phosphatase as same as the renowned drug metformin [5]. It also reduces oxidative stress and preserves and protects beta cell mass. As a result, glucose enters the cells through GLUT4 expression via facilitated diffusion, and signalling molecules such as AMPK and CaMKII are vital in regulating cellular GLUT4 expression [6,7].

The above-mentioned potential health benefit of Q.U. and β-crt are mainly dependent on their bioavailability, but unfortunately, they possess low aqueous solubility and, upon oral administration, undergoes rapid degradation in the gastric environment [8,9,10]. Hence, developing the appropriate novel delivery system would help overcome the formulation hitches and augment its therapeutic effectiveness. Therefore, developing the appropriate novel delivery system would help overcome the formulation problems and increase its therapeutic value. In such attempts, the lipid-based drug delivery system that enhances the solubility and bioavailability of hydrophobic components is the right choice to deliver hydrophobic bioactives like β-crt [11,12,13]. Nanoemulsion (N.E.) also called as colloidal delivery system comprises of biphasic system and is least influenced by physical and chemical variants, comprising temperature and pH [14,15]. Accordingly, these features make the nanoemulsion system an ideal choice to potentially encapsulate the bio-active compounds using various lipids and surfactants to improve their solubility, absorption and cellular uptake [15]. Ultrasonication is a high-energy method (HEM) that produces N.E. with smaller droplet sizes, PDI with prolonged stability requires a relatively lesser surfactant than other HEM such as homogenisation and microfluidization. Fabrication of N.E. with good stability using the ultrasonication technique depends on various parameters, and optimising these parameters becomes indispensable to achieve stable N.E.

The Box Behnken Design is a multivariant optimisation technique based on a three-level incomplete factorial design [16,17]. BBD is beneficial over the other Response Surface Method (RSM) for the quadratic model owning the efficient design with fewer experimental runs and a cost-effective approach [18]. Many studies also suggested that RSM is a beneficial technique for N.E. optimisation [19].

Recently, our previous study attempted to develop an ultrasonically fabricated Quercetin Nanoemulsion (QU-NE) to improve oral bioavailability and enhance its therapeutic efficacy, wherein the QU-NE significantly improved bioavailability, regulated blood glucose levels and body weight, reduced oxidative stress markers and protected the liver and kidney insulin resistance [18].

It was also reported that a combination of bioactives comprising glycyrrhizin and thymoquinone polymeric nanoformulation was developed and successfully improved diabetic conditions in rats [20]. Another study has emphasised the potential benefits of combining bioactives and bioactives with synthetic antidiabetic drugs in a novel delivery system for managing diabetes [21].

Therefore, with these justifications, it was hypothesised that combining β-crt and Q.U. in a nanoemulsion-based delivery system would provide potential combinatorial benefits in Diabetes management. Thus, the present investigation was designed to develop and optimise ultrasonically assisted β-crt and Q.U. Nanoemulsion using 3-factor 3-level Box–Behnken design (BBD). The second objective was to evaluate the antidiabetic properties of βC-NE alone and in combination with QU-NE in the streptozotocin-induced diabetic rat model.

## 2. Materials and Methods

### 2.1. Materials

β-Carotene (β-crt) was procured from Sisco Research Laboratories (CAS-7235-40-7), Hyderabad, India. Capmul MCM C8 (Glyceryl Monocaprylate) (CAS-26402-26-6) and Acconon MCM C8 EP/NF (Polyoxyethylene Caprylic/Capric Glycerides) were kindly gifted by Abhitec Corp, Garnet Valley, PA, USA. Gelucire 44/14 (Lauroyl polyoxyl-32 glycerides N.F.) was a gift sample from Gattefosse, India. Streptozotocin (STZ) was procured from Sigma-Aldrich, Bangalore, India. The kit employed for biochemical parameter estimation was purchased from Spinreact, Spain. The Millipore water was employed for formulation and analysis. The analytical grade chemicals, reagents, and solvents were employed in the current study.

### 2.2. Assessment of β-Crt Solubility in Nanoemulsion Components

A surplus amount of βC was added to 1 mL of different oils, including gingelly oil-GGO; rice bran oil-RBO; soya bean oil-SBO; Canola oil-CNO; olive oil- OLO; ethyl oleate-EO and Capmul- C.P.Surfactants including Labrafil-LFIL; Gelucire 44/14-GLE; tween 20-T20; span 20-S20 and span 80-S80. Co-surfactants include propylene glycol-PG, Transcutol-P-TP and Acconon—ACN. The samples were then stored in an isothermal shaker for 72 h at 25 ± 02 °C to attain equilibrium. The samples were centrifuged at 6000 rpm (REMI, Maharashtra, India, Ultracentrifuge) for 10 min. The collected supernatant was filtered using a 0.22 μm membrane filter, and the β-crt content was determined at 461 nm by UV-visible spectrophotometer 1700, Shimadzu, Columbia, MD, USA [18].

### 2.3. Selection of Surfactants

Oil emulsification is essential to obtain a stable emulsion. Therefore, surfactants, including the labrafil M 2125 CS, tween 20, Aconon and gelucire 44/14, were screened for capmul mcm c8 (oil) emulsifying ability. To 2.5 mL of respective surfactant solutions (15% *w*/*v*), 5 μL of oil was added incrementally and vortexed repetitively until it turned cloudy. The surfactant, which could emulsify the maximum oil, was selected, and results were expressed in percentages [18].

### 2.4. Selection of Co-Surfactants

The co-surfactants consisting of Propylene Glycol (P.G.), Transcutol P (T.P.) and Aconon (AcN) were selected in a 1:1 ratio with Gelicire 44/14 and tested for their ability to yield a larger nanoemulsion range through Creating ternary phase diagrams (TPDs). As described previously, the Oil and Smix (surfactant and co-surfactant) were evaluated in fifteen possible combinations [18].

### 2.5. Effect of Smix Mass Ratio

The Smix in the different weight ratios 1:1, 1:2, 1:3, 2:1 and 3:1 were selected, and their impact on nanoemulsion formation was studied using ternary phase diagrams (TPD). Initially, the Smix ratios were selected with an increasing surfactant concentration while the co-surfactant concentration was kept constant and vice-versa. TPD was constructed using the aqueous titration method, adding water to each weight ratio of oil and Smix, followed by vortexing at 25 °C [18,22].

### 2.6. Preparation of β-Crt-Nanoemulsion(βC-NE)

The specified amount of β-crt was dissolved in the organic phase, consisting of the stated amount of Capmul, gelucire and Acconon. The crude emulsion was obtained by homogenising (T-25 Ultraturrax, IKA, Bengaluru, India). The organic phase with dissolved β-crt was added to the aqueous phase at 5000 rpm for 5 min. The obtained emulsion was later processed with ultra-sonication (Vibra cell VCS 750-220, Sonics, Newton, CT, USA) at pre-set amplitude and time.

### 2.7. Optimisation of βC-NE by Box-Behnken Design (BBD)

Three factor three level Box Behnken design (BBD) was applied to optimise nanoemulsion. The quadratic response surfaces represented by the second-order polynomial model were analysed using Design Expert^®^ (Version 10.0 Stat-Ease Inc., Minneapolis, MN, USA). The independent variables selected were Smix ratio (A), % Amplitude (B) and Sonication time (C), and the globule size, PDI and % E.E. responses selected were the selected responses (Table 1).

### 2.8. Determination of Globule Size (G.S.), Polydispersibility Index (PDI) and Zeta Potential (Z.P.)

The G.S., PDI, and Z.P. of βC-NE were measured using a dynamic light scattering (DLS) technique employing Zetasizer ZS90 (Malvern, Worcestershire, UK). The measurement was performed as described previously at 25 °C.

### 2.9. Determination of Entrapment Efficiency (% E.E.)

The β-crt -loaded N.E. was centrifuged (REMI Electrotechnik Ltd., Vasai, India) at 12,000 rpm for 20 min. Later, the supernatant obtained was clarified through a 0.22 µm membrane filter. The β-crt was quantified using the method previously described with slight modification [23]. Briefly, HPLC (Shimadzu HPLC system-LC20C, I prominence series) with a UV-Visible detector consisting of a C18 column (5 µm particle size ODS, 150 mm × 4.6 mm) at 30 °C ± 2°C was employed. The mobile phase is constituted of methanol, tetrahydrofuran (THF) and water in a 70:23:7 ratio. The injection volume was 20 µL, and the flow rate was adjusted to 1.2 mL/min and detected at 465 nm. The Lab-solution was used to process the chromatograms, and the E.E. was computed and stated in percentage [24,25].

### 2.10. Impact of Storage Condition on G.S. and PDI

The optimised βC-NE was stored in transparent glass vials away from light at 4 and 25 °C for a period of 45 days. The globule size and PDI have been analysed accordingly on 0, 7, 15, 30 and 45th day [18].

### 2.11. In Vitro β-Crt Release Studies

The release of β-crt pure drug (βC-PD) and βC-NE was studied by dialysis, employing double distilled water with 1% tween 20 as a release medium according to the previously described [18]. The previously soaked dialysis tube of molecular weight cut-off (MWCO) 12 KD (Himedia Laboratories Pvt, Ltd., Mumbai, India.) was selected and the βC-NE and βC-PD suspended in Na-CMC (Sodium carboxymethylcellulose) were added and made impenetrable at both ends. The dialysis tube was then suspended in release media (50 mL) and positioned on a stirrer at 100 rpm and 37 °C ± 2 °C. The samples were withdrawn at frequent intervals (15, 30, 60, 120, 180, 240, 300, 360, 420, 480, 540, 600, 660 and 720 min) and replenished with new release media to maintain the sink condition. The samples were then diluted with methanol and quantified for β-crt release from N.E. using the HPLC method.

### 2.12. Morphological Characterisation by Transmission Electron Microscope (TEM)

The surface morphological investigation and globular-size confirmation were performed using Transmission electron microscopy (TEM-JEOL/JEM 2100, Tokyo, Japan). Adjustments were made such that the series of bright field imaging at increasing magnification and diffraction modes revealed the form and size of the βC-NE. A drop of βC-NE was suitably diluted in distilled water (1:100), and a drop of the diluted nanoemulsion was placed on a copper grid and stained with uranyl acetate for 0.5 min, and was examined under the microscope after drying [26].

### 2.13. Animals

Albino Wistar rats of about 200–250 g in weight were procured from Biogen Laboratory Animal Facility (Bengaluru, India). The rats were housed under a controlled environment of 12 h—12 h light cycles and at the temperature of 23 °C for 15 days with free access to water. The Institutional ethical committee approval for all the experimental procedures was obtained from the Institutional Animal Ethics Committee (IAEC) JSS College of Pharmacy, JSS AHER (proposal number: IAEC/JSSCPM/345/2019).

### 2.14. In-Vivo Pharmacokinetics Study

After acclimatisation, twelve rats were randomly allocated into group-A and group B (*n* = 6) and fasted overnight with access only to water. The group-A animals were administered with β-crt pure drug (βC-PD) of 30 mg/kg dispersed in 7.2 pH phosphate buffer, and group-B animals were administered with optimised βC-NE equivalent to 30 mg/kg in a single oral dose. 300 µL of blood samples were collected from the tail vein into a centrifuge tube containing 100 µL of 3% sodium citrate solution at 0 h (before dosing), 1 h, 2 h, 4 h, 8 h, 16 h, 24 h and 48 h after of dosing. The plasma from the collected blood samples was separated by centrifugation at 8000 rpm for 10 min. The HPLC method, as described above, was employed to analyse the β-crt in plasma, and the pharmacokinetic parameters were computed by the non-compartmental model using Pumas-Julia Computing software v.1.1.0 [27].

### 2.15. Induction of Diabetes and Animal Grouping

Streptozocin (STZ) at 40 mg/kg body weight for used for diabetes induction in a method described previously [14]. Later, the rats were segregated into five groups (*n* = 6) Normal (without any induction), Control (STZ-induced diabetes), Standard (Diabetes induced and metformin-treated), βC-NE (diabetes + βC-NE treated) and βC-NE+QU-NE (diabetes + combination treated). The normal and control group received 1.5 mL of distilled water daily, the standard received metformin of 250 mg/kg B.W. daily, the βC-NE group received 15 mg/kg B.W., and βC-NE+QU-NE received 7.5 mg/kg of βC-NE + 6.25 mg/kg of QU-NE. All animals were subjected to 21 days of treatment with careful monitoring throughout the experimental period.

### 2.16. Measurement of Body Weight (B.W.), Blood Glucose Level (BGL), and Oral Glucose Tolerance Test (OGTT)

The animals’ B.W. was measured using a calibrated weighing machine, whereas the BGL of all the rats was measured using a blood glucose meter (ACCU-CHEK, Indianapolis, IN, USA) employing test strips as per manufacturer’s instruction by the tail-vein method on 0, 7, 14 and 21st day [23]. On the 10th and 20th days of the study, the animals were administered glucose (2 mg/kg B.W.) after overnight fasting. The blood glucose level was monitored at 0, 20, 60, 90 and 120 min after glucose administration to determine the oral glucose tolerance [18].

### 2.17. Analysis of Biochemical Parameters, Tissue and Oxidative Stress-Markers

The biochemical parameters including Serum Alanine Aminotransferase (ALT), Aspartate aminotransferase (AST), total cholesterol (T.C.), total glycerides (T.G.), high-density lipoproteins (HDL), low-density lipoproteins (LDL) were determined after 21 days of treatment by the kit method (Spinreact, Girona, Spain) using Varioskan LUX Multimode Microplate Reader, Thermofisher Scientific (Waltham, MA, USA). The serum creatinine (cre) and blood urea nitrogen (BUN) were measured using kits from Elabscience, Houston, TX, USA. Whereas the VLDL was measured according to the previously established procedure. Further, the lipid peroxidation (LPO) and superoxide dismutase (SOD) levels were estimated using the tissue homogenate from the isolated liver [18].

### 2.18. Histopathological Analysis of Pancreas and Liver Tissue

The Collected liver and pancreatic tissue samples were cleaned and fixed using a formalin solution (10%) before embedding in paraffin. The sections were subjected to microscopic examination after staining with haematoxylin and eosin.

### 2.19. Statistical Methods

All the data are expressed as the mean ± standard deviation (S.D). Statistical significance was evaluated using analysis of variance (ANOVA) employing GraphPad 8.0 Software Inc., San Diego, CA, USA).

## 3. Results

### 3.1. Selection of Nanoemulsion Components

**Figure 1:**
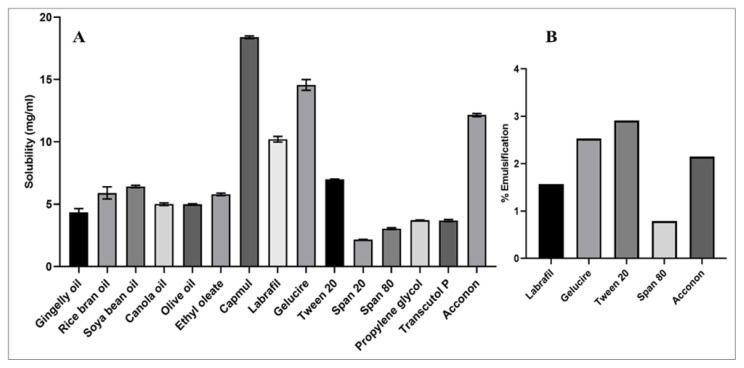
(**A**) solubility analysis of β-crt in various Nanoemulsion constituents; (**B**) % capmul Emulsification by chosen surfactants.

The β-crt solubility in various oils, surfactants and co-surfactants was determined (Figure 1A). β-crt exhibited higher solubility in capmul (18.39 ± 0.11mg/mL) than other oils, followed by Gleucire (14.56 ± 0.43 mg/mL) among surfactants, and acconon MCM C8 (12.15 ± 0.12 mg/mL) among co-surfactants. Higher drug solubility in the oil phase enables higher drug loading in the Nanoemulsion (N.E.) system and influences the preparation volume for therapeutic dose delivery. Thus, oil selection is crucial in N.E. preparation [28]. The surfactants play a significant role in N.E. as emulsifiers but are known to cause gastrointestinal toxicity [28]. Hence, non-ionic were selected in the study owing to their reduced toxicity, ability to form stable N.E. at lower energy and, consequently, improve the formulation’s stability, biocompatibility, ability to withstand the alterations in the ionic and pH environment and provide improved in vivo stability [9,19,29]. The oil in water (o/w) N.E. is formed above the HLB of 10. As a result, the present series of surfactants (Gelucire, Tween 20, Span 20, S80 and Acconon) with a wide range of HLB values (10–16.7) were screened for their emulsifying ability along with β-crt solubility of oral N.E. preparation. Tween 20 and Gelucire 44/14 emulsified the capmul to a greater extent, with the Tween 20 showing the highest emulsification attributed to its higher HLB value (Figure 1B). The co-surfactants are employed to obtain the stable N.E. at lower surfactant concentration preparation [30].

**Figure 2:**
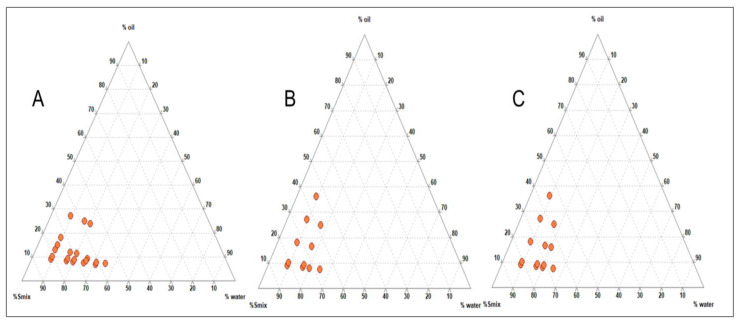
Ternary Phase Diagram depicting the nanoemulsion region obtained for capmul and tween 20 with various co-surfactants: (**A**) Propylene glycol; (**B**) Transcutol P and (**C**) Acconon at 1:1 ratio.

**Figure 3:**
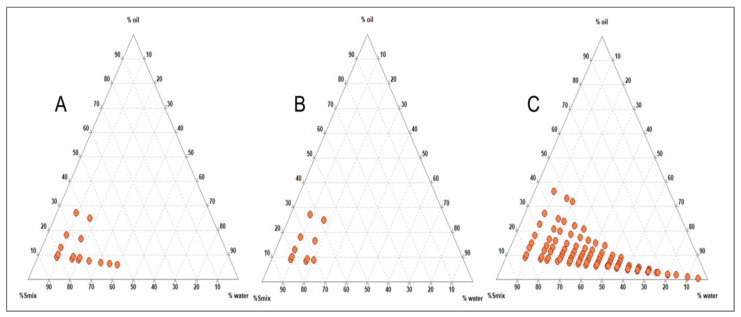
Ternary Phase Diagram depicting the nanoemulsion region obtained for capmul and gelucire with various co-surfactants: (**A**) Propylene glycol; (**B**) Transcutol P and (**C**) Acconon at 1:1 ratio.

Various co-surfactants were screened in combination with surfactants, initially with Tween 20 and later with Gleucire, separately to produce a larger N.E. region. The aqueous titration method was employed, and TPDs were developed using Triplot 4.1 software, (Informer Technologies, Inc., Rheinland-Pfalz, Germany). It was observed that Tween 20, in combination with other co-surfactants, did not yield a satisfactory N.E. region (Figure 2A–C), but Gelucire 44/14 produced a significantly larger N.E. region with Acconon than other co-surfactants (Figure 3A–C). Thus, capmul as oil phase, Gelucire as surfactant and Acconon as cosurfactant were selected for the study.

### 3.2. Effect of Smix Mass Ratio

**Figure 4:**
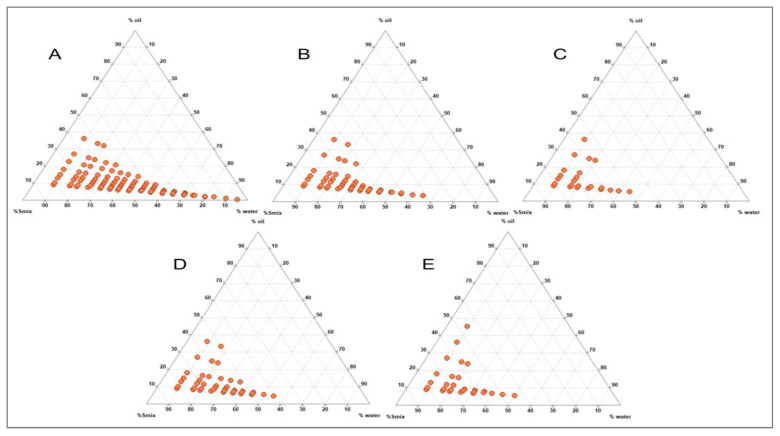
Ternary Phase Diagram depicting the effect of Smix (Gelucire and Acconon) with capmul at various ratios: (**A**) 1:1; (**B**) 1:2; (**C**) 1:3; (**D**) 2:1 and (**E**) 3:1.

The impact of the Smix mass ratio was evaluated further for optimising the nanoemulsion system (Figure 4). A larger nanoemulsion region was observed in Figure 4A, where surfactant Gelucire and Acconon were employed at a 1:1 ratio. However, the nanoemulsion region decreased as the co-surfactant concentration in the Smix increased to 1:2 (Figure 4B) and 1:3 (Figure 4C). Hence no further increment in co-surfactant was evaluated. A similar decrease in the nanoemulsion region was observed when the surfactant concentration in the Smix was increased to 2:1 (Figure 4D) and 3:1 (Figure 4E). Hence, it was confirmed that Gelucire and Acconon at a 1:1 ratio could achieve optimum emulsification with capmul which could be correlated to the HLB value of the Smix composition. Gelucire has the HLB of 11, and Acconon HLB value is 12. The HLB value of the Smix ranged from 11.25 to 11.75. The larger N.E. region was formed at Smix, 1:1 (Figure 4A), indicating that the HLB value of 11.5 is optimum to achieve emulsification of capmul and produce a larger nanoemulsion region. In the nanoemulsion region, a phase diagram shown towards the water-rich apex could be diluted significantly [30]. In the current study, the organic phase composition (consisting of oil, surfactant and co-surfactant) was kept constant at 10% of the total formulation, wherein the oil phase ratio was constant at one, and the Smix ratio was varied from 5 to 9.

### 3.3. Optimisation of βC-NE by Box Behnken Design (BBD)

The BBD-based experimental trials for globule size (nm), polydispersibiliIndex (PDI), and entrapment efficiency (E.E.) were 136.9 ± 12.45 to 274.8 ± 17.65 nm, 0.131 ± 0.02 to 0.402 ± 0.05, and 65.44 ± 1.26 to 79.12 ± 2.85% respectively (Table 2).

The responses were studied employing design expert-11 software, wherein the quadratic polynomial model was suggested for globule size (*p* < 0.001) and entrapment efficiency (*p* < 0.001), and the PDI was independent of any variables PDI (*p* > 0.05). The ANOVA analysis, fit statistics, and regression coefficient values are shown in Table 3, and the regression equations obtained are summarised in Table 4. The larger F-value and smaller *p*-value confirmed the significant levels of each variable. (Table 3).

**Figure 5:**
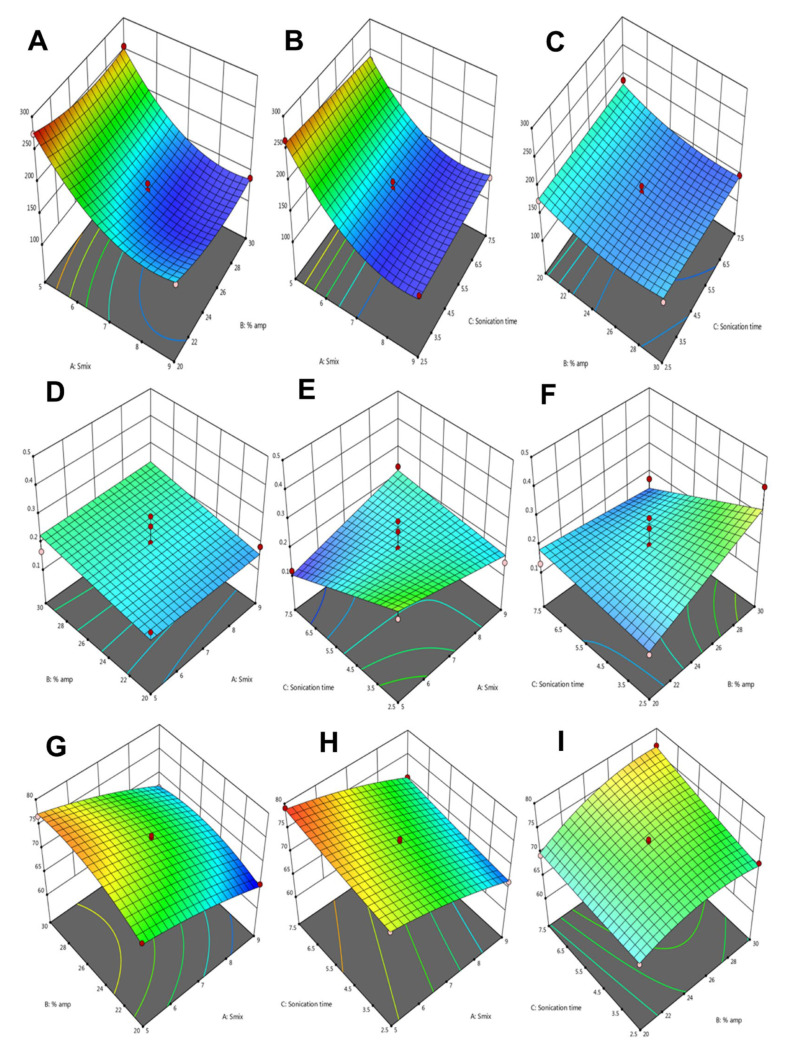
3D response surface graphs illustrating the interaction effect for the globule size of βC-NE: (**A**) Smix and % amplitude; (**B**) Smix and Sonication time; (**C**) % amplitude and sonication time; (**D**) Smix and % amplitude; (**E**) Smix and Sonication time; (**F**) % amplitude and sonication time; (**G**) Smix and % amplitude; (**H**) Smix and Sonication time, and (**I**) % amplitude and sonication time.

An increased Smix ratio reduced the globule size with a correspondingly higher amplitude due to increased shear and cavitation forces, as depicted in Figure 5A and the regression equation. In addition, a higher Smix ratio reduced the system’s interfacial tension, resulting in a smaller globule size. The effect of Smix and sonication time are represented in Figure 5B. As the Smix and sonication time was increased, the βC-NE globule size was found to decrease. The effect of % amplitude and sonication time is depicted in Figure 5C. The globule size was reduced at a higher amplitude owing to the high shear generation, which breaks the larger globules into smaller ones.

Though no variables significantly impacted PDI, it was found to be high at lower Smix and sonication time; however, it was reduced with an increase in Smix and sonication time (Figure 5D,E). As depicted in Figure 5F, the PDI increased with an increase in % amplitude.

The % E.E. was primarily affected by Smix, % amplitude, and sonication time. The % E.E. increased with an increase in the % amplitude and sonication time but showed a decrease with an increase in Smix (Figure 5G–I).

The quadratic equations obtained (Table 4) suggested that a higher Smix with an optimum % amplitude and sonication time would yield a smaller globule with an optimum PDI and a higher entrapment efficiency. Further, the processing conditions were verified by formulating βC-NE under suggested optimum conditions. Thus, BBD was successfully employed to study the effects of Smix, % amplitude, and sonication time on globule size, PDI, and % E.E.

The suggested optimised conditions were validated by developing βC-NE under suggested conditions with desirability 1.0 (Table 5). The obtained βC-NE and its responses were in agreement with that of the suggested values wherein the globule size was 153.1 ± 12.25 nm, PDI was 0.200 ± 0.04 and 73.25 ± 3.25% E.E. (Table 5). The negative zeta potential could be attributed to the dissociation of fatty acid adsorbed or negatively charged ions at the interface.

### 3.4. Impact of Storage Condition on G.S. and PDI of βC-NE

**Figure 6:**
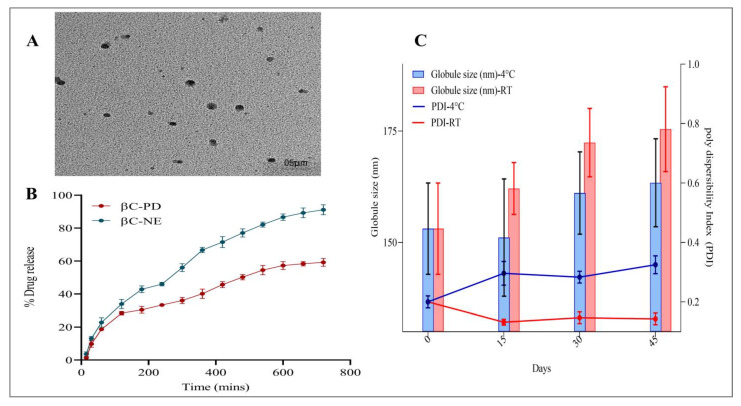
(**A**) TEM image of optimised βC-NE; (**B**) In-vitro drug release profile of βC-NE and βC-PD; (**C**) Effect of storage condition on globule size and PDI of optimised βC-NE.

Nanoemulsions are thermodynamically unstable formulations. Hence, determining the stability of the N.E. becomes essential. In the present study, the effect of storage conditions on G.S. and PDI was measured at 4 and 25 °C for 45 days (Figure 6C). It was observed that the G.S. and PDI tend to increase from the 0th day to the 15th day, except for G.S. showing slightly decreased G.S. on day 15. The growth in G.S. and PDI could be attributed to the globules’ coalescence behaviour [29]. However, there was no visible separation or changes at the end of the 45th day in both storage conditions. The increase in G.S. at the end of the 45th day was less than 10%, and the PDI was well within 0.35.

### 3.5. In Vitro Drug Release Study of βC-NE

The dialysis method was employed in the current study. The in-vitro release of βC-NE and βC-PD was studied using double distilled water. Because β-crt is less soluble in water and physiological fluids, the release medium was modified using 1% *v*/*v* of T20, as reported in earlier studies [28,31,32]. The cumulative release of βC-NE was superior to the βC-PD with approximately 91.10 ± 1.02% of β-crt being released from NE in 12 h. In contrast, only 59.22 ± 1.35% of β-crt was released from the βC-PD (Figure 6B). Also, βC-NE exhibited sustained-release properties as the β-crt in N.E. had to release from the lipid and surfactant matrices through diffusion. Thus, the developed N.E. delivery system can help encapsulate β-crt and other bioactive compounds, sustain their release rate from the formulation and improve their absorption and bioavailability in-vivo. Furthermore, the study agreed with the previously conducted studies [13,33].

### 3.6. Morphological Characterisation of βC-NE by TEM

The morphological analysis of βC-NE was examined using TEM micrographs. The nanoemulsion droplets were spherical with sizes varying in 5 μm scale. The obtained data agreed with the droplet size analysis of the βC-NE formulation (Figure 6A).

### 3.7. In Vivo Pharmacokinetic Studies of βC-NE and βC-PD

**Figure 7:**
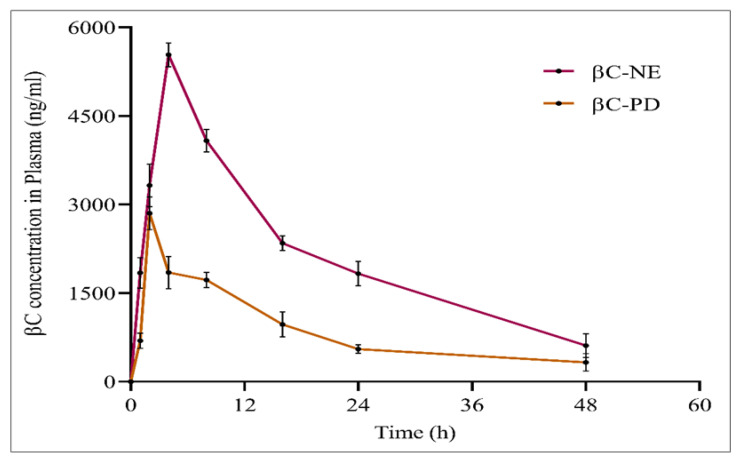
Plasma concentration-time profile of optimised βC-NE and βC-PD.

The HPLC technique was adopted pharmacokinetic study of βC-NE and βC-PD oral administration. The mean plasma concentration-time profile (Figure 7) indicates the maximum drug concentration (Cmax) achieved and the time taken to reach Cmax (Tmax). The further pharmacokinetic parameters were analysed using a non-compartmental model by employing Pumas–Julia Computing software (Table 6).

The βC-NE and βC-PD, after single oral administration, reached the maximum drug concentration (Cmax) of 5536.90 ± 203.486 ng/mL and 2851.64 ± 274.03 ng/mL after 4 and 2 h, respectively The Cmax of βC-NE was ~2 folds higher than βC-PD, and the tmax of βC-NE was also delayed compared with that of βC-PD, demonstrating the effectiveness of N.E. system in improving the β-crt absorption and sustained the release. Moreover, the mean resident times (MRTs) of βC-NE was 1.5 fold higher than βC-PD, which could be due to the sustained release of β-crt. The NE system also reduced the β-crt distribution by 1.5 times than βC-PD. The AUC^0−t^ and AUC^0−∞^ were increased by 4.8 and 5.5 times, respectively.

The above parameters indicate that βC-NE had improved the oral bioavailability owning to increased drug absorption and residence time. The results obtained correlated well with the previously reported studies [18,34].

The NE system improves bioavailability by improving intestinal permeability by Smix and reducing gastric degradation and clearance. The smaller globule size facilitates the effective intestinal uptake of β-crt, thus avoiding the first-pass metabolism [35]. Furthermore, the Smix could have increased the permeability or enhanced the adhesion to the G.I. tract wall [36]. The reduced bacterium exposure and enzymatic degradation by successfully encapsulating β-crt into the lipid matrix also contribute to extended in vivo contact with the intestinal wall, owning to the possible adhesion of N.E. to the mucosal surface of intestinal tissue [37]. Moreover, the N.E. system influencing the prolonged circulation of β-crt could be attributed to the extended systematic residence of β-crt [35].

### 3.8. Influence of βC-NE and βC-NE+QU-NE on Body Weight (B.W.)

**Figure 8:**
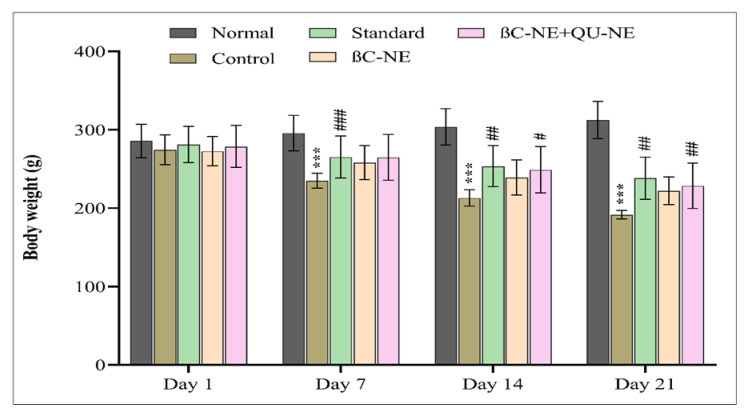
Impact of βC-NE and βC-NE+QU-NE on body weight (B.W.). The data represent mean ± standard deviation. The significance was measured using one-way ANOVA. *** *p* < 0.001 vs. Normal group. # *p* < 0.05, ## *p* < 0.01 and ### *p* < 0.001 vs. Control group (*n* = 6).

The B.W. of the rats was measured on the 1, 7, 14 and 21st day of the treatment. It was observed that the control group’s body weight significantly reduced on the 7th day compared to the normal group. on the contrary, the standard and βC-NE and βC-NE+QU-NE treated groups significantly inhibited weight loss. A similar trend was observed on the 14th, and 21st day, wherein the control group showed further decrease in body weight compared to normal groups. However, it was noticeable on the 21st day that the βC-NE+QU-NE and the standard group had significantly inhibited bodyweight reduction (Figure 8). The results agreed with previous studies wherein Quercetin Nanoemulsion (QU-NE) exhibited similar inhibition in weight loss [18].

### 3.9. Influence of βC-NE and βC-NE+QU-NE on Blood Glucose Level (BGL)

**Figure 9:**
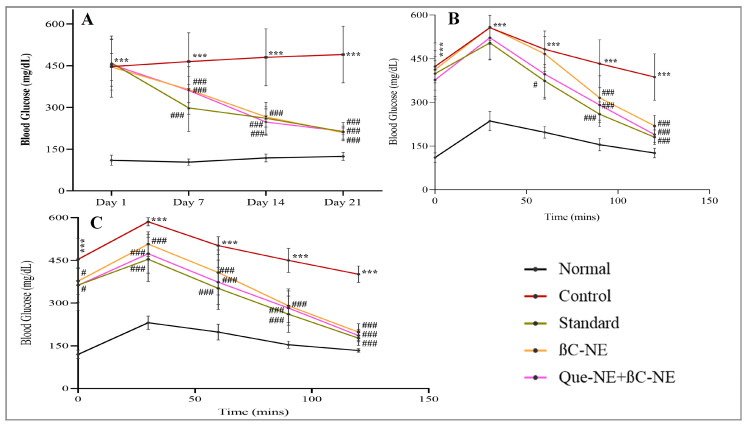
Impact of βC-NE and βC-NE+QU-NE on: (**A**) blood glucose level; (**B**) oral glucose tolerance test on the 10th day; (**C**) Oral glucose tolerance test on the 20th day. The data represent mean ± standard deviation. The Significance was calculated employing one-way ANOVA: *** *p* < 0.001 vs. Normal group. # *p* < 0.05, and ### *p* < 0.001 vs. Control group (*n* = 6).

The results of BGL measurement revealed that the treatment groups had significantly higher BGL on day 01 compared to the normal group indicating hyperglycaemic condition. However, it was notable that the other treated groups had significantly inhibited the rise in blood glucose levels. the control group showed gradual increase, whereas the standard and treated groups significantly reduced the elevated BGL on the 7th day, and on the 14th and 21st day, all the treatment groups had significantly lesser BGL in comparison to that of the control group. It was evident that the βC-NE and βC-NE+QU-NE treated groups prominently managed BGL similarly to standard at the end of the 21st day. (Figure 9A).

### 3.10. Influence of βC-NE and βC-NE+QU-NE on Oral Glucose Tolerance Test (OGTT)

The OGTT performed on days 10 and 20 exhibited that glucose tolerance was significantly impaired in the control group compared to the normal group. On the 10th day, the βC-NE+QU-NE treated group had significant tolerance from the 30th minute. Moreover, all other groups were also found to be significant glucose tolerance compared to the control group (Figure 9 B). On the 20th day, all the treatment groups showed significant glucose tolerance from 30th to 120 min. The βC-NE+QU-NE group showed remarkable glucose tolerance on the 10th and 20th days (Figure 9B,C). Similar to that of Q.U., β-crt also modulates the lipids and carbohydrates metabolism, resulting in improved functioning of pancreatic β-cells and conditions of hyperglycemia [38,39].

### 3.11. Influence of βC-NE and βC-NE+QU-NE on Lipid Levels

**Figure 10:**
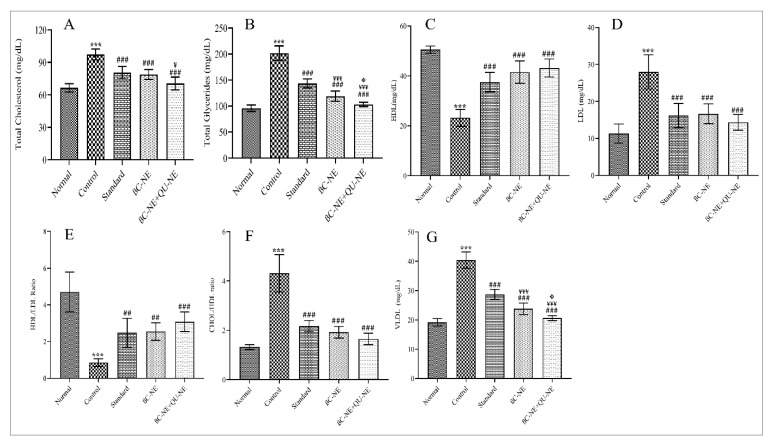
Impact of βC-NE and βC-NE+QU-NE on lipid profiles: (**A**) Total Cholesterol (T.C.); (**B**) Total Glycerides (T.G.); (**C**) High-density lipoproteins (HDL); (**D**) Low-Density Lipoproteins (LDL); (**E**) HDL/LDL ratio; (**F**) CHOL/HDL ratio, and (**G**) Very Low-Density Lipoproteins (VLDL). The data represent the mean ± standard deviation (*n* = 6). Significance was calculated employing one-way ANOVA. *** *p* < 0.001 vs. Normal group. ## *p* < 0.01, ### *p* < 0.001 vs. Control group ¥ *p* < 0.05 and ¥¥¥ *p* < 0.001 vs. Standard group Φ *p* < 0.05 vs. BC-NE group.

The lipid levels revealed that the control group significantly increased T.C., T.G., LDL and VLDL levels compared to the normal group, while the all other treated groups significantly inhibited the increase. The control group showed reduced HDL levels, whereas, the standard, βC-NE and βC-NE+QU-NE treated groups improved HDL levels significantly. At the same time, the βC-NE+QU-NE group had a noticeable impact on lipid levels compared to the βC-NE treated group (Figure 10). Hyperlipidemia is the foremost cause for various diabetes and its complications. In the current work, βC-NE, especially the βC-NE+QU-NE group, had a potential impact on lipid levels compared to βC-NE alone, demonstrating that the combinatorial effect of Q.U. and β-crt to decrease or prevent other diabetic complications by preventing hyperlipidemia (Figure 10). The obtained results were in agreement with earlier studies where, a nanoemulsion-based delivery system successfully improved the therapeutic effectiveness by improving the stability and bioavailability of the bioactive agent compared to traditional delivery systems [18,40].

### 3.12. Influence of βC-NE and βC-NE+QU-NE on Tissue Injury and Oxidative Stress Markers

**Figure 11:**
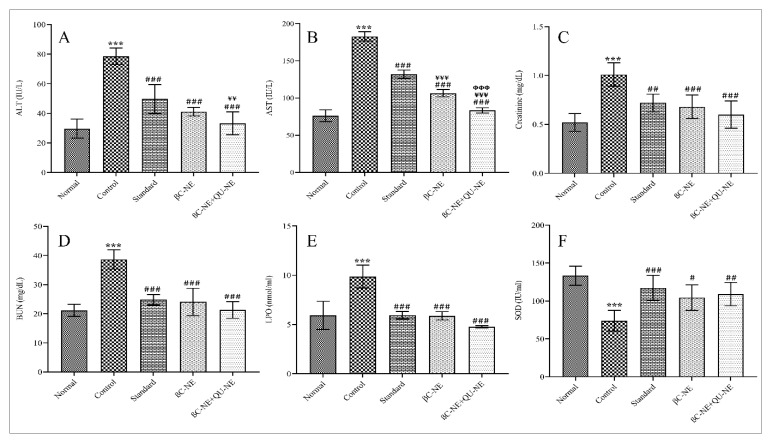
Impact of βC-NE and βC-NE+QU-NE on tissue injury and oxidative stress markers. (**A**) Alanine aminotransferase (ALT); (**B**) Aspartate aminotransferase (AST); (**C**) creatinine; (**D**) Blood–Urea–Nitrogen (BUN); (**E**) lipid peroxidation; (**F**) Superoxide dismutase (SOD). The data represent mean ± standard deviation. Significance was calculated employing one-way ANOVA. *** *p* < 0.001 vs. Normal group. # *p* < 0.05, ## *p* < 0.01 and ### *p* < 0.001 vs. Control group. ¥¥ *p* < 0.01 and ¥¥¥ *p* < 0.001 vs. Standard group. ΦΦΦ *p* < 0.001 vs. BC-NE group (*n* = 6).

AST and ALT are the markers of liver damage, while BUN and creatinine are the markers of Kidney. Higher values signify the tissue damage initiation through diabetes-induced oxidative stress [3]. Quercetin protects hepatocytes through enhanced lipophagy [39]. Moreover, the Qu exert nephroprotection by protein kinase C inhibition and down-regulation of transforming growth factor 1 (TGF-1) [38]. In contrast, β-crt is reported to ameliorate liver fibrosis by inhibiting iNOS and NF-B in-vivo [39,40]. In the present study, increased levels of AST, ALT, BUN, creatinine and LPO were observed in the control group, while the other treated groups showed a significant reduction in the levels of these markers. Similarly, SOD levels in the control group were reduced but improved in the treated groups (Figure 11). Overall, βC-NE+QU-NE showed slightly better protection than βC-NE, proving that Q.U. and β-crt acting by different biochemical mechanisms and having improved bioavailability by nanoemulsion delivery could have augmented the therapeutic efficacy in inhibiting or preventing injury or diabetic complications.

### 3.13. Histopathology Hepatic and Pancreatic Cells

**Figure 12:**
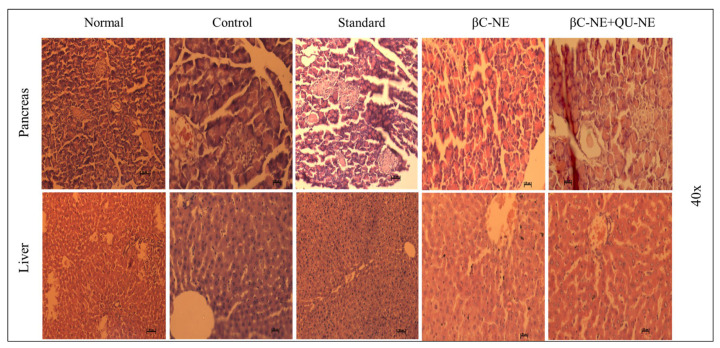
Histopathological images of pancreatic and liver tissues after 21-day treatment (scale bar = 50 µm).

The disorganised β-islets cells and groups of inflammatory cells were evident in the control group. The pancreatic β-cells displayed darkly-stained deteriorated nuclei and the dilated hepatic portal vein due to apoptosis in the liver. In contrast, the Normal group showed a characteristic pancreatic cell structure with β-islets cells surrounded by exocrine acini, with a regular β-cell population and an organised pancreas with decreased darkly stained inflammatory cells. Alongside normalised liver, prominent hepatocytes were observed in the standard group. The βC-NE treated group displayed fewer β-cells with slight damage in the pancreatic tissue. However, the typical structure of Liver tissue with prominent hepatocytes was witnessed. The group treated with βC-NE+QU-NE showed re-organised islets of Langerhans with increased β-cells and normalised liver structure with prominent hepatocytes. The darkly stained nuclei with the radially arranged central veins in hepatocytes were visible. Thus, it was prominent that the βC-NE+QU-NE had delivered significant pancreas and hepatocyte protection (Figure 12).

## 4. Conclusions

The current research work focused on fabricating ultrasonically assisted β-carotene nanoemulsion (βC-NE) employing capmul, Gelucire 44/14 and acconon. The effect of Surfactant ratio (Smix), % amplitude, and sonication time on globule size, Polydispersibility Index, and entrapment efficiency of βC-NE and recognised the optimised conditions (7% Smix at 25% amplitude for 5 min of ultrasonication). The optimised βC-NE showed significantly higher bioavailability compared to βC-PD. The βC-NE at 15 mg/kg B.W. exhibited therapeutic activity in diabetic rats for 21 days. The βC-NE, in combination with QU-NE, exhibited superior antidiabetic potential compared to βC-NE alone by regulating the body weight and blood sugar level and inhibiting raised lipid levels. Additionally, the βC-NE and QU-NE significantly suppressed the tissue injury and oxidative stress markers. Thus, the combinational therapy of β-carotene and Quercetin in a nanoemulsion-based delivery system exhibiting superior therapeutic efficiency can be a potential substitute therapy in managing diabetes.

## Figures and Tables

**Table 1:** Independent variables and their corresponding levels for βC-NE optimisation by BBD.

Character	Independent Variables	Actual Levels at Coded Factor Levels		
		−1	0	1
A	S_mix_ ratio *	5	7	9
B	% Amplitude	20	25	30
C	Sonication time (mins)	2.5	5.0	7.5

* Smix ratio- Surfactant and co-surfactant ratio.

**Table 2:** BBD for β-NE with independent variables and responses.

Run	Independent Variables			Responses		
	Smix Ratio	% Amp	Sonication Time	Globule Size (nm)	PDI	EE (%)
1	5	25	2.5	263.7 ± 16.86	0.269 ± 0.02	75.12 ± 1.08
2	9	20	5	163.7 ± 11.23	0.185 ± 0.03	65.44 ± 1.26
3	7	25	5	151.1 ± 15.43	0.296 ± 0.02	73.08 ± 0.78
4	7	30	2.5	155.0 ± 10.02	0.402 ± 0.05	70.72 ± 1.56
5	7	25	5	143.1 ± 12.25	0.131 ± 0.02	72.52 ± 2.02
6	5	30	5	255.7 ± 14.85	0.167 ± 0.03	76.68 ± 1.86
7	5	25	7.5	234.4 ± 19.92	0.117 ± 0.05	79.12 ± 2.85
8	7	20	2.5	175.4 ± 11.23	0.133 ± 0.02	68.76 ± 1.12
9	7	30	7.5	148.5 ± 10.86	0.172 ± 0.03	76.6 ± 1.56
10	7	25	5	165.0 ± 09.14	0.138 ± 0.02	72.92 ± 2.33
11	9	25	2.5	139.8 ± 08.44	0.147 ± 0.04	66.48 ± 2.42
12	5	20	5	274.8 ± 17.65	0.199 ± 0.02	72.6 ± 1.61
13	7	20	7.5	190.4 ± 15.25	0.131 ± 0.03	69.48 ± 2.84
14	9	25	7.5	136.9 ± 12.45	0.231 ± 0.04	70.32 ± 3.23
15	7	25	5	153.1 ± 13.56	0.200 ± 0.01	73.84 ± 1.82
16	9	30	5	138.8 ± 14.22	0.176 ± 0.02	67.12 ± 1.22
17	7	25	5	152.5 ± 11.45	0.260 ± 0.02	74.24 ± 1.47

**Table 3:** ANOVA analysis of optimisation of βC-NE by BBD.

Source	Response 1 Droplet Size (nm)		Response 2 PDI		Response 3 %EE	
ANOVA ANALYSIS						
	*f*-Value	*p*-Value	*f*-Value	*p*-Value	*f*-Value	*p*-Value
Model	54.80	0.0001	1.88	0.181	49.46	0.0001
A-S_mix_	353.26	0.0001	0.005	0.944	282.90	0.0001
B-% Amplitude	19.76	0.0030	0.005	0.9449	53.39	0.0002
C-Sonication time	0.9825	0.3546	2.67	0.133	50.55	0.0002
AB	0.1177	0.7416	0.031	0.862	2.79	0.1386
AC	2.44	0.1624	3.31	0.098	0.0124	0.9144
BC	1.62	0.2441	3.09	0.109	12.91	0.0088
A^2^	98.24	0.0001	-	-	4.53	0.0708
B^2^	12.32	0.009			36.53	0.0005
C^2^	0.0005	0.9827			0.2795	0.6134
Lack of fit					1.10	0.4450
Fit statistics						
R^2^	0.9860		0.5296		0.9845	
Adjusted R^2^	0.9680		0.2473		0.9646	
Adequate Precision	21.95		5.35		25.312	
Regression Coefficient values						
Intercept	152.96		0.1973		73.32	
A-S_mix_	−56.17		−0.0016		−4.27	
B-% Amplitude	−13.29		0.0336		1.86	
C-Sonication time	−2.96		−0.0375		1.80	
AB	−1.45		0.0057		−0.6000	
AC	6.60		0.0590		−0.0400	
BC	−5.37		0.0570		1.29	
A^2^	40.83		-		−0.7450	
B^2^	14.46		-		−2.12	
C^2^	−0.0925		-		0.1850	

**Table 4:** Regression equations of the responses for optimised βC-NE in coded terms.

**Response 1 Droplet size**	= + 152.96 − 56.17 × A − 13.29 × B − 2.96 × C − 1.45 × AB +6.60 × AC − 5.37 × BC + 40.83 × A^2^ + 14.46 × B^2^ − 0.0925 × C^2^
**Response 2 PDI**	= + 0.1973 − 0.0016 × A + 0.0336 × B − 0.0375 × C + 0.0057 × AB + 0.0590 × AC − 0.0570 × BC
**Response 3% E.E.**	= + 73.32 − 4.27 × A +1.86 × B +1.80 × C − 0.6000 × AB − 0.0400 × AC + 1.29 × BC − 0.7450 × A^2^ − 2.12 × B^2^ +0.1850 × C^2^

**Table 5:** The optimised condition for βC-NE: Predicted and experimental values.

**Factors**	**Independent Variables**	**Actual Levels**	
A	S_mix_ ratio	7.0	
B	% amplitude	25.0	
C	Sonication time (mins)	5.0	
**Responses**	**Predicted** **values**	**Experimental values**	**Desirability**
Droplet size (nm)	152.96	153.1 ± 12.25	1.0
PDI	0.197	0.200 ± 0.04	
E.E. (%)	73.32	73.25 ± 3.25	

**Table 6:** The Pharmacokinetic parameters of βC-NE and βC-PD.

Sl.No	Pharmacokinetic Parameter	βC-PD	βC-NE
1	T _max_ (h)	2 ± 0.0	4 ± 0.0
2	C_max_ (ng/mL)	2851.64 ± 274.03	5536.90 ± 203.486 **
3	C_48_ (ng/mL)	160.01 ± 38.93	612.06 ± 48.187 **
4	T_1/2_ (h)	22.54 ± 7.84	37.96 ± 7.74 *
5	AUC _0–48_ (ng/h/mL)	19,325.32 ± 864.26	94,325.65 ± 1387.50 **
6	AUC_0–∞_ (ng/h/mL)	23,345.09 ± 4231.32	125,784.35 ± 12,546.55 **
7	Ke (h^−1^)	0.04 ± 0.00	0.15 ± 0.00 *
8	Vd (mL)	15,478 ± 3267.44	6634.67 ± 824.80 *
9	Cl (mL/h)	487 ± 57.23	97.02 ± 7.67 *
10	MRT (h)	29.32 ± 9.22	45.36 ± 8.96 *

Data represented are mean ± S.D., *n* = 6. The Pharmacokinetic parameters of βC-NE were significant from βC-PD at * *p* < 0.05 and ** *p* < 0.005.

## Data Availability

Not applicable.
